# Supplementary material for: The six steps of the complete F1-ATPase rotary catalytic cycle
Source: Nat Commun. 2021 Aug 3;12:4690. doi: 10.1038/s41467-021-25029-0 (PMC8333055; doi:10.1038/s41467-021-25029-0)
Supplement: Supplementary file 1 — Supplementary information [file 41467_2021_25029_MOESM1_ESM.pdf]

SUPPLEMENTARY INFORMATION

The six steps of the complete F_1-ATPase rotary catalytic cycle

Meghna Sobti et al.

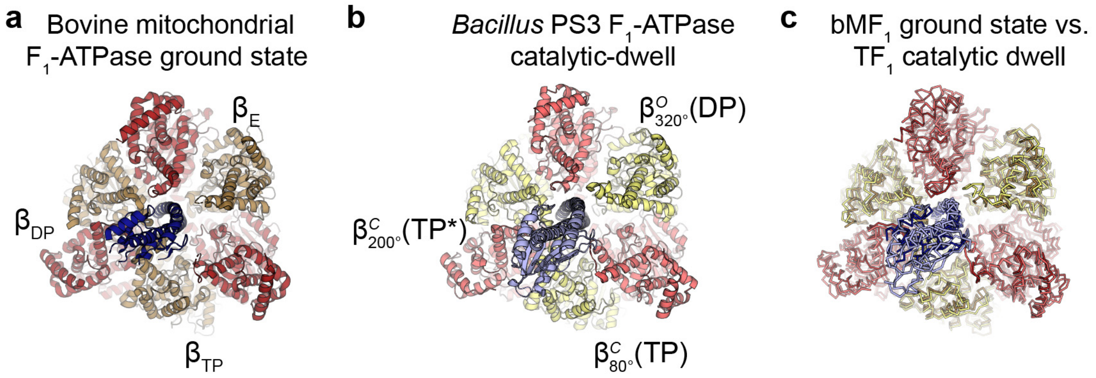

Supplementary Figure 1: Comparison of the catalytic-dwell conformation with the bovine mitochondrial F_1-ATPase ground state. (**a**) bMF_1 ground state crystal structure (pdb2JDI[1]); subunits α in dark red, β in "sand" and γ in dark blue. (**b**) TF_1 catalytic-dwell cryo-EM structure (from this study); subunits α in light red, β in light yellow and γ in light blue. (**c**) Superposition of bMF_1 ground state crystal structure (darker colors) with TF_1 catalytic-dwell cryo-EM structure (lighter colors). The structures show a similar overall fold (RMSD of 1.6 Å) in a similar rotational dwell/state.

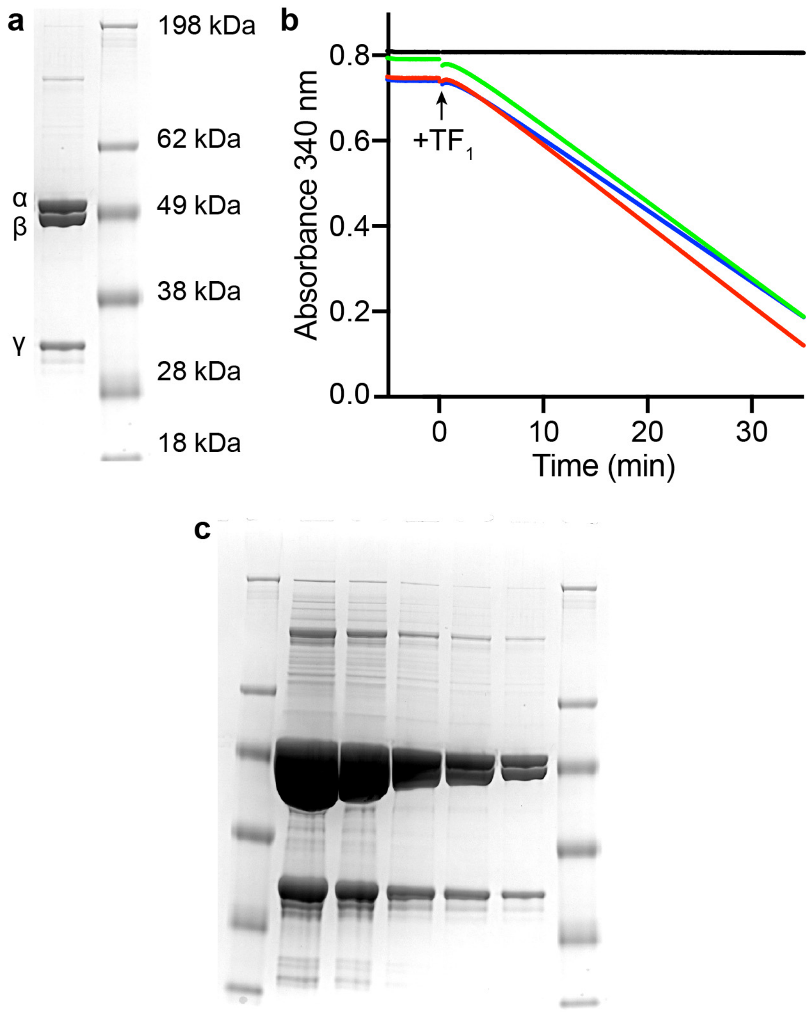

Supplementary Figure 2: SDS PAGE and ATPase activity of TF_1(βE190D). (**a**) SDS PAGE gel of purified TF_1(βE190D). *Left lane;* TF_1(βE190D). *Right lane;* SeeBlue marker (Thermo Fisher). (**b**) ATP regeneration assays of TF_1(βE190D) (red, green and blue) and control (black – no TF_1(βE190D) added) show that the enzyme has a steady state specific activity of 0.3 U/mg (comparable to the published value of ~0.4 U/mg[2]), where one unit of activity is defined as producing 1 μmol of ADP per min. 10 μg of TF_1(βE190D) added to a 1 ml reaction volume in a cuvette with 1 cm pathlength at 25°C. 6.22 Millimolar extinction coefficient of NADH at 340 nm used to calculate coupled ATP turnover. The experiment was repeated three times (red, green and blue data points) with similar results. (**c**) Uncropped raw image of gel in panel **a**.

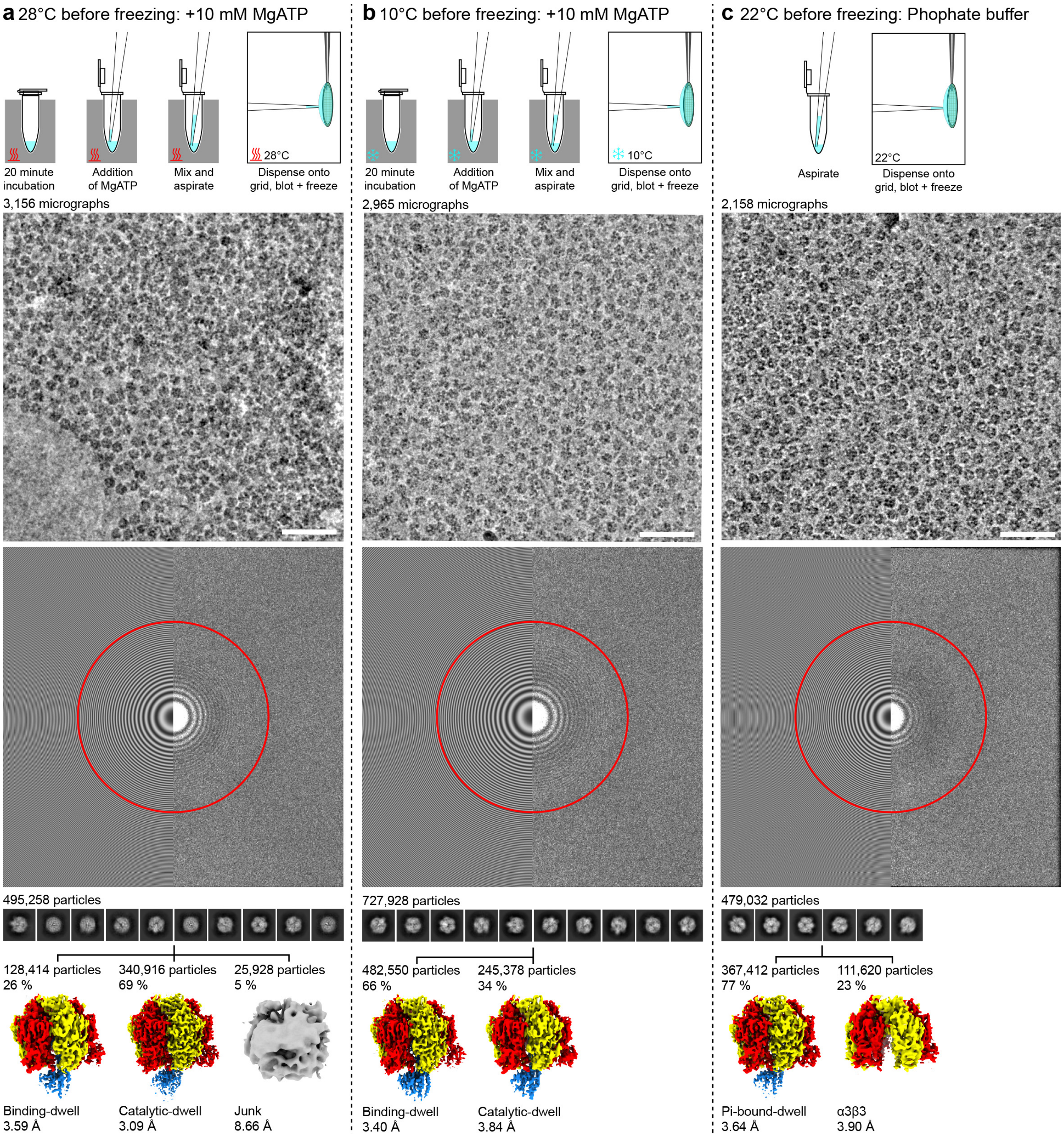

Supplementary Figure 3: Sample preparation, data collection and data processing. (**a**) 28°C + MgATP prior to plunge freezing. (**b**) 10°C + MgATP prior to plunge freezing. (**c**) 22°C in phosphate buffer prior to freezing. *Top:* schematic of sample preparation. A thermocycler was used to heat or cool the sample prior to freezing and a Vitrobot was used to control the temperature of the grid prior to plunging freezing. *Middle:* sample electron micrograph (white scale bar is equivalent to 50 nm) and power spectrum of sample (red circle at 3 Å resolution). *Bottom:* processing flowchart showing 2D classification and heterogeneous sorting[3]. The experiment was performed twice, once during screening at 200 kV (data not shown) and once at 300 kV (data in this figure), with similar results.

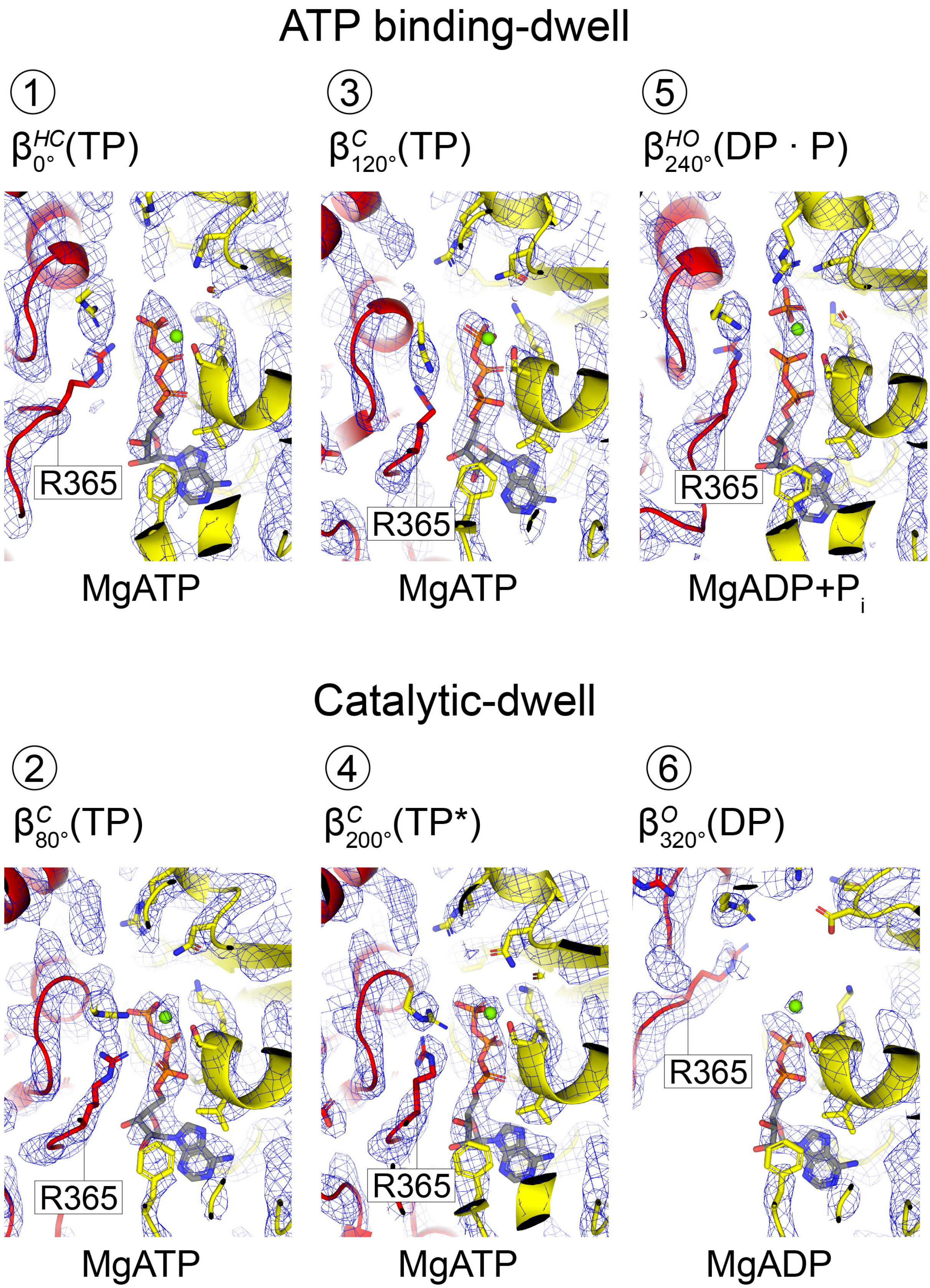

Supplementary Figure 4: Nucleotide binding sites. Cryo-EM maps of the β subunit nucleotide binding sites. Same as Fig. 3 bottom panel, but larger format.

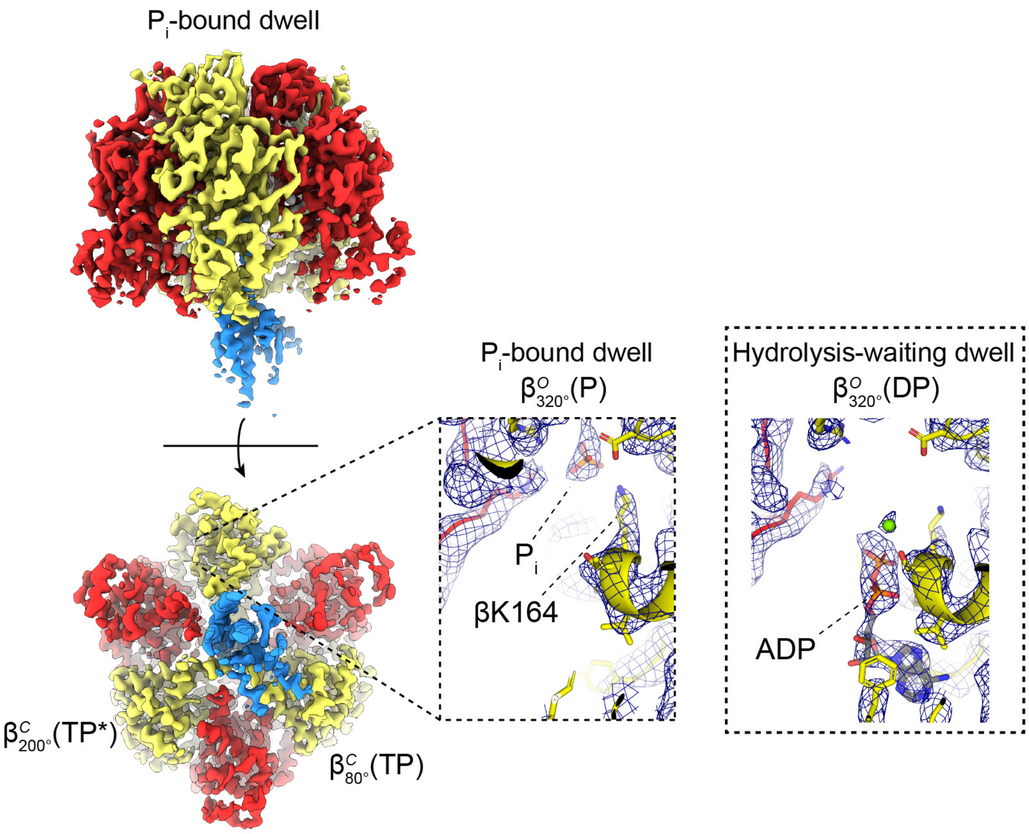

Supplementary Figure 5: Cryo-EM structure of the P$_i$-bound-dwell. The P$_i$-bound-dwell is in the same rotational state as the catalytic-dwell and contains P$_i$ in the $\beta^{O}_{320°}$ site. Cryo-EM map shown as surface viewed perpendicular and from the membrane. Subunits α in red, β in yellow and γ blue. Views of the nucleotide binding sites of the P$_i$-bound- and catalytic-dwells shown for comparison; cryo-EM map shown as blue mesh, and the atomic model shown as cartoon and sticks with CPK coloring (same view as bottom panel of Fig. 3).

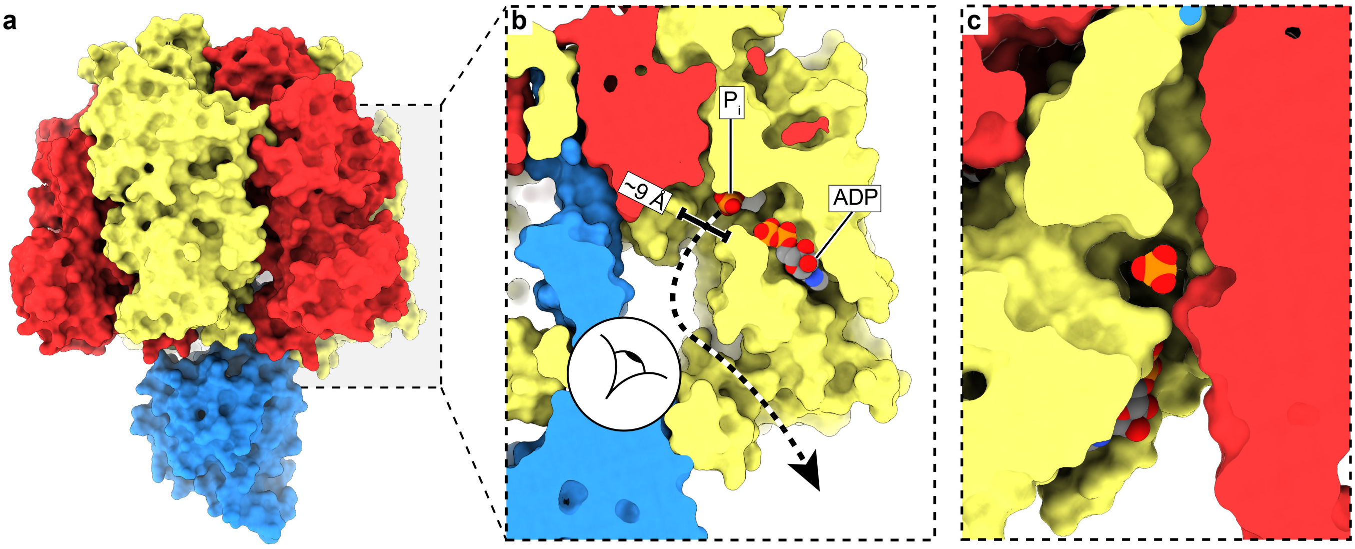

Supplementary Figure 6: The P_i alternative exit tunnel. Surface representation of the P_i-bound-dwell cryo-EM structure, to show the possible alternative exit tunnel. Figure is similar layout to Fig. 5. (**a**) Overall P_i-bound-dwell structure shown as surface. (**b**) Zoomed in section of the P_i-bound-dwell with the ADP molecule from the catalytic-dwell $\beta_{320°}$ site docked and shown as spheres with CPK coloring. (**c**) Close-up view looking down the alternative exit tunnel (viewpoint shown in panel **b** with eye pictogram).

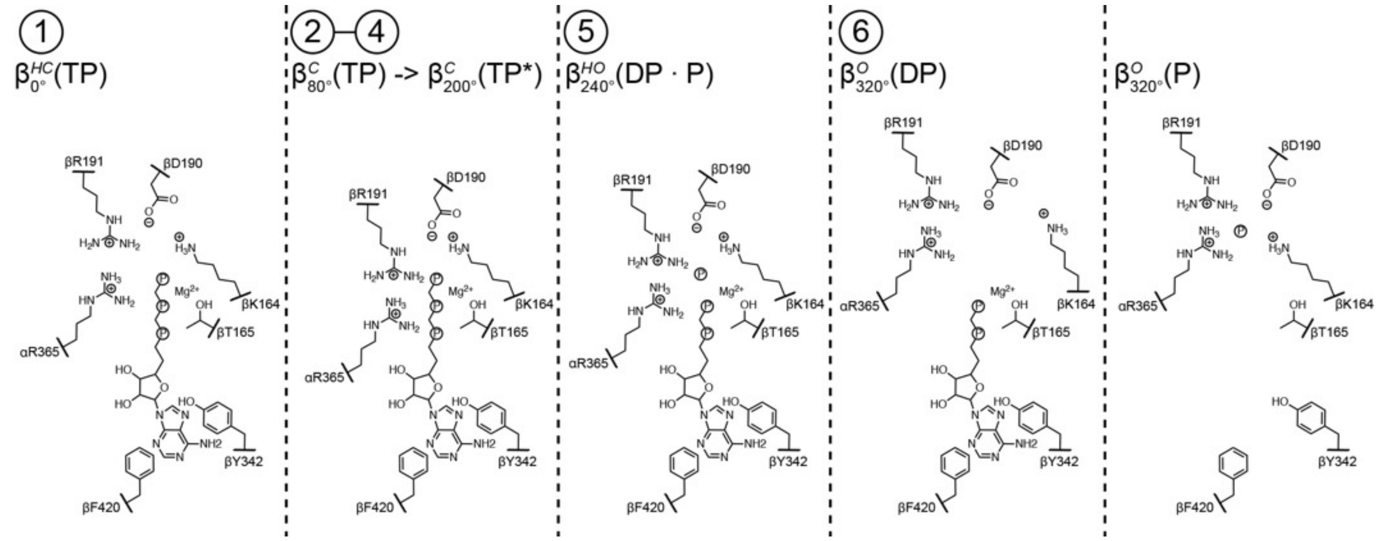

Supplementary Figure 7: Schematics of the β nucleotide binding sites. Simplified chemical representations of the catalytic binding sites, made with ChemDraw (PerkinElmer). βY342 and βF420 hold the adenosine ring of ATP. ATP binding causes αR365, βK164 and βR191 to close onto the γ-phosphate. ATP hydrolysis liberates the γ-phosphate that is coordinated by αR365, βK164 and βR191. Phosphates are shown as the letter P within a circle without oxygens to aid clarity.

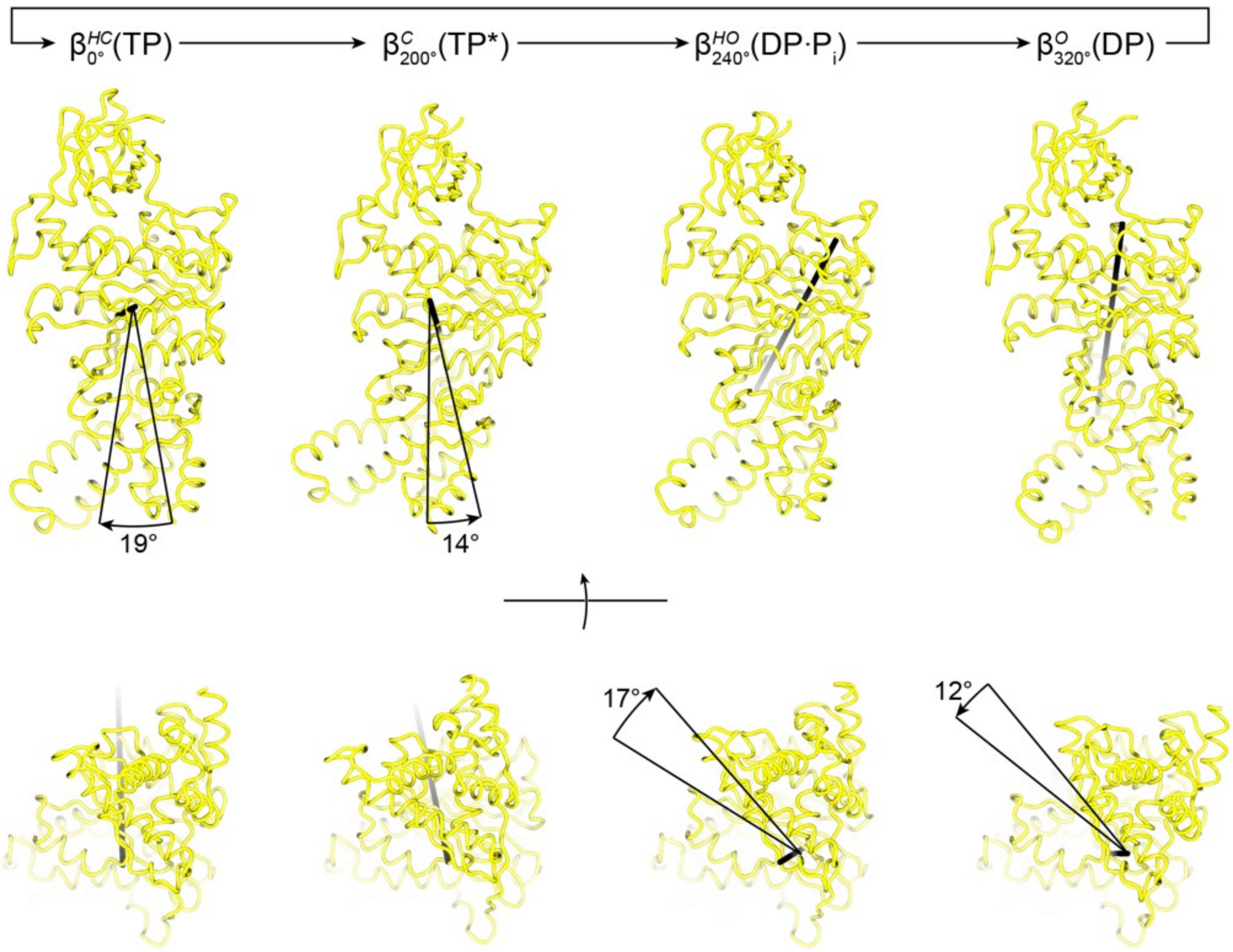

Supplementary Figure 8: The transitions from open to closed and closed to open have two movements with different rotation axes. β subunits superposed on the N-terminal β barrel, and rotation axes of the rigid body defined by residues 129-180 and 327-470 shown as a black vector. Rotation axes and rotations angels calculated with ccp4mg[4]. The angle between the rotation axes for open→half closed and half closed→closed is 59°. The angle between rotation axes for closed→half open and half open→open is 50°.

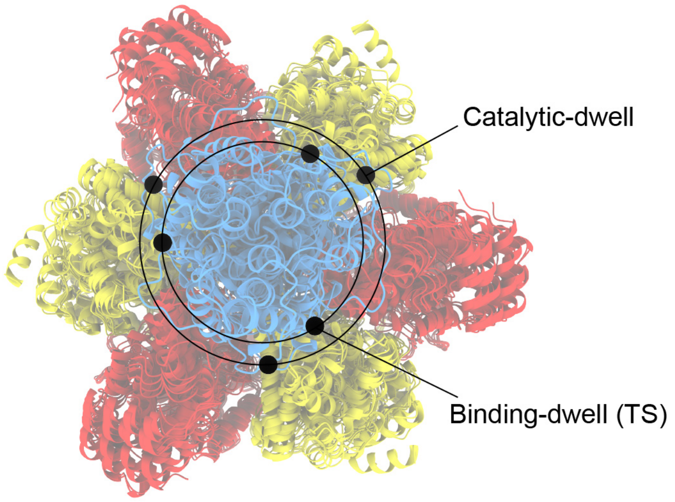

Supplementary Figure 9: The γ subunit precesses or wobbles as it rotates. Superposition of the six rotational positions (on residues β2-82) of the enzyme shows that the two dwell states have different rotation radii. Protein shown as cartoon with black spheres for the Cα of γC112.

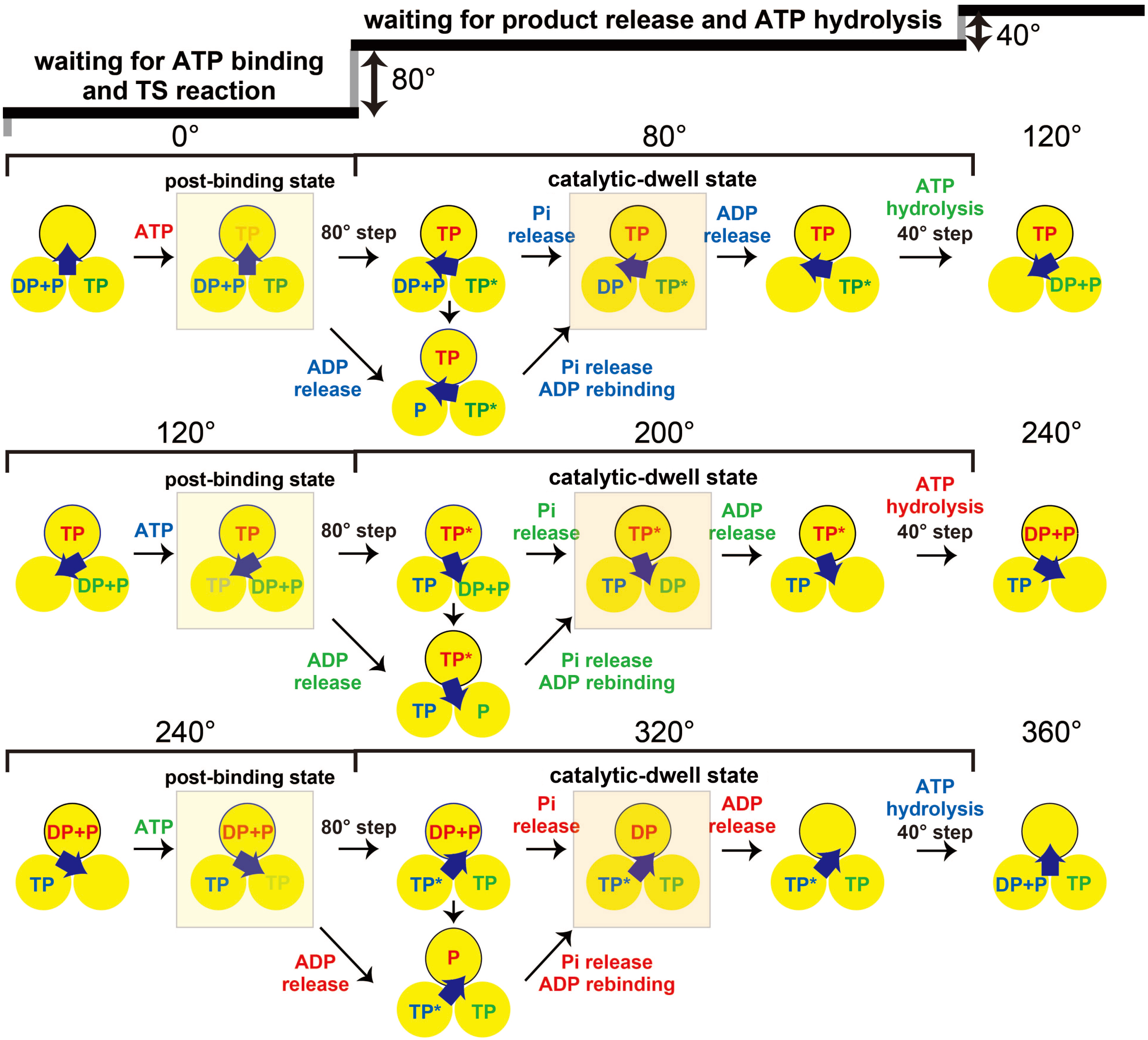

Supplementary Figure 10: Possible models of chemo-mechanical coupling of TF$_1$ (βE190D). Yellow circles represent the chemical states of catalytic sites in β subunits. The central blue arrows represent the orientations of γ subunits. TP, DP and P indicate ATP, ADP and P$_i$, respectively. TP* indicates ATP in catalysis. Green, yellow and orange squares indicate the states considered from the cryo-EM structures obtained in this study. 0° is defined as the ATP binding angle for the catalytic site outlined in black. In these models, ATP (red) bound at 0° is hydrolyzed to ADP and P$_i$ at 200°. Three possible schemes are shown for product release. (i) ADP (red) is released during the 80° substep from 240° to 320° and then P$_i$ is released at 320°. (ii) ADP (red) is released at 320° after the 80° substep from 240° to 320° and then P$_i$ is released at 320°. (iii) P$_i$ (red) is released at 320° after the 80° substep from 240° to 320° and then ADP (red) is released at 320°. Other catalytic sites also obey the same reaction scheme offset by 120° and 240°.

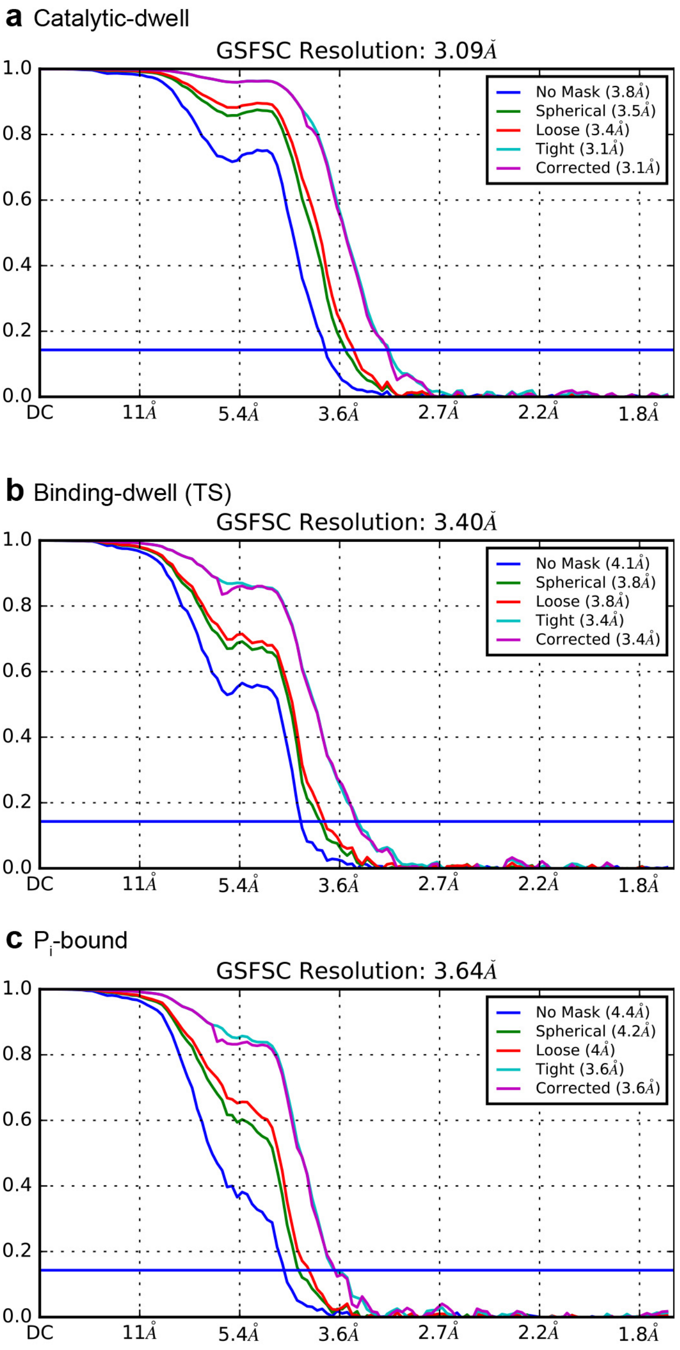

Supplementary Figure 11: FSC curves. FSC outputs from cryoSPARC[3]. (**a**) catalytic-dwell, (**b**) binding-dwell (TS) and (**c**) Pi-bound-dwell.

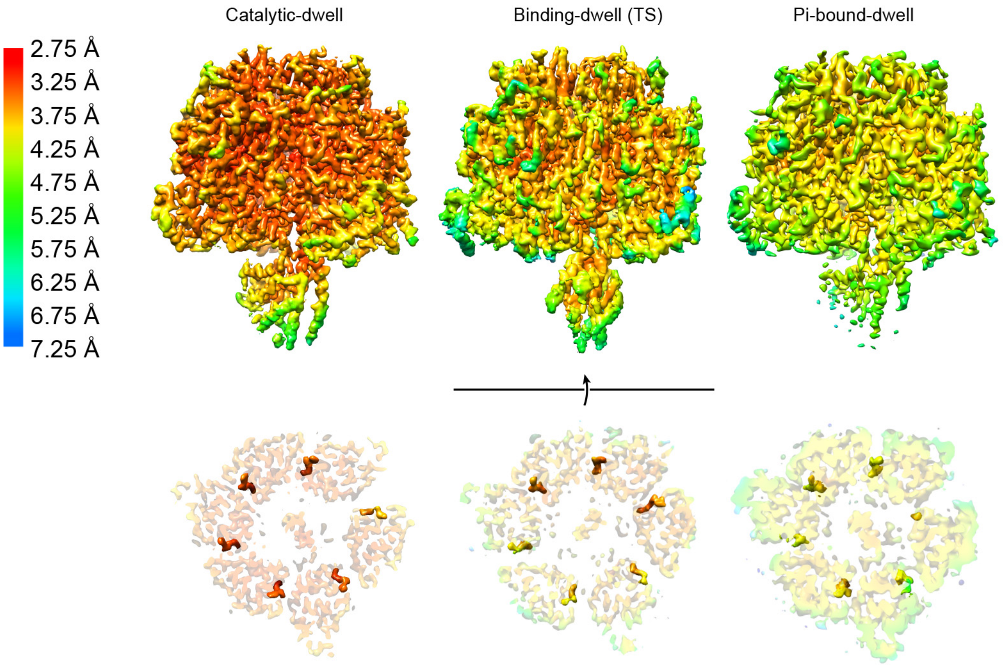

Supplementary Figure 12: Local resolution estimates. Cryo-EM maps colored with local resolution estimates for the catalytic-dwell, binding-dwell (TS) and Pi-bound-dwell. Implemented in cryoSPARC[3] and displayed using UCSF Chimera[5]. *Left*; color legend to describe resolution estimate. *Top*; full maps shown from the side. *Bottom*; section through TF_1 with the density corresponding to the nucleotides/ions highlighted.

Supplementary Table 1: Cryo-EM data collection, refinement and validation statistics.

	#1 Catalytic-dwell (EMDB-23116) (EMDB-24139) (PDB 7L1R)	#2 Binding-dwell (TS) (EMDB-23115) (EMDB-24138) (PDB 7L1Q)	#3 P_i-bound-dwell (EMDB-23117) (EMDB-24140) (PDB 7L1S)
**Data collection and processing**			
Magnification	60,000	60,000	60,000
Voltage (kV)	300	300	300
Electron exposure ($e^-/Å^2$)	50	50	50
Defocus range (μm)	0.5-1.5	0.5-1.5	0.5-1.5
Pixel size (Å)	0.84	0.84	0.84
Symmetry imposed	C1	C1	C1
Initial particle images (no.)	495,258	727,928	479,032
Final particle images (no.)	340,916	482,550	367,412
Map resolution (Å) 0.143 FSC threshold	3.1	3.4	3.6
**Refinement**			
Initial models used (PDB codes)	6N2Y, 4XD7	#1 Catalytic-dwell	#1 Catalytic-dwell
Model resolution (Å) 0.5 FSC threshold, masked/ unmasked	3.2/3.2	3.9/3.9	4.0/4.3
Map sharpening *B* factor ($Å^2$)	108	115	146
Model composition			
Non-hydrogen atoms	24,199	24,204	24,176
Protein residues	3,118	3,118	3,118
Ligands	12	13	11
B factors ($Å^2$)			
Protein	161	68	161
Ligand	152	69	145
R.m.s. deviations			
Bond lengths (Å)	0.012	0.008	0.012
Bond angles (°)	1.784	1.278	1.832
Validation			
MolProbity score	1.78	1.63	1.90
Clashscore	3.87	2.65	4.37
Poor rotamers (%)	3.32	2.58	4.07
Ramachandran plot			
Favored (%)	96.71	96.13	96.55
Allowed (%)	3.25	3.70	3.29
Disallowed (%)	0.03	0.16	0.16

References:

1 Bowler, M. W., Montgomery, M. G., Leslie, A. G. & Walker, J. E. Ground state structure of F_1-ATPase from bovine heart mitochondria at 1.9 Å resolution. *J Biol Chem* **282**, 14238-14242, doi:10.1074/jbc.M700203200 (2007).

2 Enoki, S., Watanabe, R., Iino, R. & Noji, H. Single-molecule study on the temperature-sensitive reaction of F1-ATPase with a hybrid F1 carrying a single beta(E190D). *J Biol Chem* **284**, 23169-23176, doi:10.1074/jbc.M109.026401 (2009).

3 Punjani, A., Rubinstein, J. L., Fleet, D. J. & Brubaker, M. A. cryoSPARC: algorithms for rapid unsupervised cryo-EM structure determination. *Nat Methods* **14**, 290-296, doi:10.1038/nmeth.4169 (2017).

4 McNicholas, S., Potterton, E., Wilson, K. S. & Noble, M. E. Presenting your structures: the CCP4mg molecular-graphics software. *Acta Crystallogr D Biol Crystallogr* **67**, 386-394, doi:10.1107/S0907444911007281 (2011).

5 Pettersen, E. F. *et al.* UCSF Chimera--a visualization system for exploratory research and analysis. *J Comput Chem* **25**, 1605-1612, doi:10.1002/jcc.20084 (2004).
