# Supplementary material for: The six steps of the complete F1-ATPase rotary catalytic cycle
Source: Nat Commun. 2021 Aug 3;12:4690. doi: 10.1038/s41467-021-25029-0 (PMC8333055; doi:10.1038/s41467-021-25029-0)
Supplement: Supplementary file 6 — Description of additional supplementary files [file 41467_2021_25029_MOESM6_ESM.docx]

Description of additional supplementary items

Title: Supplementary Movie 1

Description: Close up of the β subunit nucleotide binding sites. Description in the top left corner denotes site being observed at that time. View as in Fig. 3 (bottom).

Title: Supplementary Movie 2

Description: Rotation of TF1. (a) viewed from the side. (b) viewed from below (the membrane). Each β subunit labelled as it cycles though the states.

Title: Supplementary Movie 3

Description: The six conformations of the β subunit. Superposed as in Fig. 4, with black arrows to show the general direction that the β subunit “foot” moves in
